# Comparative genomics of drug resistance in *Trypanosoma brucei rhodesiense*

**DOI:** 10.1007/s00018-016-2173-6

**Published:** 2016-03-14

**Authors:** Fabrice E. Graf, Philipp Ludin, Christian Arquint, Remo S. Schmidt, Nadia Schaub, Christina Kunz Renggli, Jane C. Munday, Jessica Krezdorn, Nicola Baker, David Horn, Oliver Balmer, Adalgisa Caccone, Harry P. de Koning, Pascal Mäser

**Affiliations:** 1 Swiss Tropical and Public Health Institute, Socinstrasse 57, 4051 Basel, Switzerland; 2 University of Basel, 4000 Basel, Switzerland; 3 Institute of Infection, Immunity and Inflammation, University of Glasgow, Glasgow, G12 8TA UK; 4 Biological Chemistry and Drug Discovery, School of Life Sciences, University of Dundee, Dow Street, Dundee, DD1 5EH UK; 5 Department of Ecology and Evolutionary Biology, Yale University, New Haven, CT USA; 6 Present Address: The University of Kent, Canterbury, Kent CT2 7NZ UK

**Keywords:** African trypanosomes, Aquaporin, RNA-binding protein, Purine permease, Pentamidine, Melarsoprol

## Abstract

*Trypanosoma brucei rhodesiense* is one of the causative agents of human sleeping sickness, a fatal disease that is transmitted by tsetse flies and restricted to Sub-Saharan Africa. Here we investigate two independent lines of *T. b. rhodesiense* that have been selected with the drugs melarsoprol and pentamidine over the course of 2 years, until they exhibited stable cross-resistance to an unprecedented degree. We apply comparative genomics and transcriptomics to identify the underlying mutations. Only few mutations have become fixed during selection. Three genes were affected by mutations in both lines: the aminopurine transporter *AT1*, the aquaporin *AQP2*, and the RNA-binding protein *UBP1*. The melarsoprol-selected line carried a large deletion including the adenosine transporter gene *AT1*, whereas the pentamidine-selected line carried a heterozygous point mutation in AT1, G430R, which rendered the transporter non-functional. Both resistant lines had lost *AQP2*, and both lines carried the same point mutation, R131L, in the RNA-binding motif of UBP1. The finding that concomitant deletion of the known resistance genes *AT1* and *AQP2* in *T. b. brucei* failed to phenocopy the high levels of resistance of the *T. b. rhodesiense* mutants indicated a possible role of *UBP1* in melarsoprol–pentamidine cross-resistance. However, homozygous in situ expression of *UBP1*-Leu^131^ in *T. b. brucei* did not affect the sensitivity to melarsoprol or pentamidine.

**Electronic supplementary material:**

The online version of this article (doi:10.1007/s00018-016-2173-6) contains supplementary material, which is available to authorized users.

## Introduction

Human African trypanosomiasis (HAT, also known as sleeping sickness) is a fatal disease caused by *Trypanosoma brucei rhodesiense* and *T. b. gambiense* in East- and West-Africa, respectively. These protozoan parasites are transmitted by tsetse flies and proliferate extracellularly in the bloodstream and lymph of their mammalian hosts, evading the adaptive immune response through antigenic variation of their variant surface glycoprotein (VSG) coat. Ultimately the trypanosomes also infest the cerebrospinal fluid, causing the ‘sleeping sickness’ syndrome of infected patients. *Trypanosoma brucei* has an approximate haploid genome size of 35 Mb, which can vary up to 25 % [1]. Excluding the kinetoplast (i.e., mitochondrial) DNA leaves a nuclear core genome of about 26 Mb, divided into 11 megabase-sized chromosomes, where the vast majority of the predicted >9000 protein-coding genes are located [2]. The treatment of HAT relies on just five drugs. Patients in the first, hemolymphatic stage are treated with suramin (*T. b. rhodesiense*) or pentamidine (*T. b. gambiense*). In the second stage, when the trypanosomes have invaded the central nervous system, melarsoprol or nifurtimox-eflornithine combination therapy (NECT; only for *T. b. gambiense*) are used [3]. These drugs are outdated, impractical, and suffer from severe adverse effects. Melarsoprol, in particular, causes unacceptable toxicity [4]. Furthermore, melarsoprol treatment failure rates of up to 30 % have been reported throughout sub-Saharan Africa [5–8]. New drugs that are safe and orally available are presently in clinical development [9]. Meanwhile, it is essential to sustain the current drugs in spite of their shortcomings, which requires an understanding of the mechanisms of drug resistance. This will also help avoid cross-resistance between current treatments and those in development.

The molecular mechanisms of drug resistance have predominantly been studied in *T. b. brucei*, which is non-pathogenic to humans and widely used as a model in molecular parasitology. A phenomenon that has been repeatedly observed is melarsoprol–pentamidine cross-resistance (MPXR), i.e., trypanosomes selected for resistance with a melaminophenyl arsenical turned out to be cross-resistant to pentamidine and vice versa [10–12]. This phenomenon was attributed to the finding that the uptake of melarsoprol and pentamidine into the trypanosomes is mediated by the same set of transporters: the aminopurine permease P2 [13, 14], encoded by the gene *AT1*, and a high-affinity pentamidine transport activity designated as HAPT1 [15, 16] recently shown to correspond to the aquagylceroporin AQP2 [17, 18]. Mutations in these transporters were described from drug-resistant *T. brucei* ssp. isolates from the field [19–21]. In the lab, MPXR was phenocopied by reverse genetics. Homozygous deletion of either *AT1* or *AQP2* resulted in resistance to both melarsoprol and pentamidine. However, the obtained resistance factors were only between 2 and 3 for melarsoprol and pentamidine in *AT1* null trypanosomes [22, 23], respectively, and 2 for melarsoprol and 15 for pentamidine in *AQP2* null mutants [17].

Here we investigate two clonal drug-resistant lines of *T. b. rhodesiense* that exhibit markedly higher levels of MPXR than observed after deletion of either *AT1* or *AQP2*. The lines *T. b. rhodesiense* STIB900-M and STIB900-P had been selected in vitro from their drug-susceptible parent *T. b. rhodesiense* STIB900 by continuous in vitro exposure to increasing concentrations of melarsoprol and pentamidine, respectively, over a period of 24 months [24]. Finally, both lines exhibited a high level of MPXR with in vitro resistance factors up to 80 (the resistance factor was defined as IC_50_ of the selected line divided by the IC_50_ of the drug-sensitive parent). This phenotype was stable in the absence of drug pressure and after passage through mice. An initial genotypic characterization demonstrated that *AT1* had been lost in STIB900-M but was still present in STIB900-P [24]. Evidently, given the high level of drug resistance, further mutations must be involved. We have performed whole genome sequencing and RNA-Seq of the parental *T. b. rhodesiense* STIB900 and its resistant derivatives STIB900-M and STIB900-P, aiming to elucidate the molecular mechanisms underlying the unprecedented level of MPXR by comparative genomics and transcriptomics.

## Materials and methods

### T. b. rhodesiense lines

*Trypanosoma brucei rhodesiense* STIB900 is a derivative of STIB704, isolated from a male patient at St. Francis Hospital in Ifakara, Tanzania, in 1981. After several passages in rodents and a cyclic passage through a tsetse fly (*Glossina morsitans morsitans*), a cloned population was adapted to axenic growth. *T. b. rhodesiense* STIB900-M and STIB900-P had been selected independently in vitro for resistance to melarsoprol and pentamidine, respectively [24]. Bloodstream-form trypanosomes were propagated in vitro as described in [20] and adapted from [25]. Cells were counted with the CASY^®^ Cell Counter system (Roche). Large numbers of trypanosomes for DNA isolation were obtained by inoculating female Naval Medical Research Institute (NMRI) mice (Harlan Laboratories) with 10^6^ trypanosomes. At peak parasitemia, the trypanosomes were harvested and separated from the blood cells on DEAE-cellulose columns [26].

### In vitro drug sensitivity

For all the STIB900 lines drug sensitivities were determined with the Alamar blue assay [27]. *T. b. brucei* B48 drug sensitivities were determined as described using a slightly modified protocol [28]. The SoftMax Pro software and Prism 5 (GraphPad) were used to calculate 50 % inhibitory concentrations (IC_50_) by non-linear regression fitting to a sigmoidal dose–response curve. All assays were performed at least three times independently, each in duplicate or triplicate. The following compounds were tested, each individually: melarsoprol (Sanofi-Aventis/WHO), eflornithine (Sanofi-Aventis), DB75 (Immtech), fexinidazole (DNDi, Geneva), nifurtimox (WHO, Geneva), suramin (Bayer), pentamidine isethionate, diminazene aceturate, phenylarsine oxide, aminopurinol, cordycepin, adenosine arabinoside, and tubercidin (Sigma). Statistical tests were performed with GraphPad Prism 5.0.

### Isolation of nucleic acids

Genomic DNA was isolated by phenol/chloroform extraction from bloodstream-form trypanosomes propagated in mice. To check for contamination with mouse DNA we performed PCR with primers for mouse glyceraldehyde-3-phosphate-dehydrogenase (GAPDH) and mouse cDNA as a positive control. For each *T. b. rhodesiense* line, about 60 µg of genomic DNA was prepared for sequencing. Total RNA was isolated from exponentially growing cultures of trypanosomes (10^6^ cells/ml) with TRIzol (Life technologies). Equal amounts of total RNA were pooled from three independent isolations, and from each pooled sample 12 µg were used for sequencing.

### Spliced leader trapping

Library preparation and RNA-Seq were performed according to the spliced leader trapping (SLT) protocol [29]. This is a modification of the standard Illumina protocol that uses the *T. brucei* 39 nt spliced leader sequence (which is a peculiarity of trypanosomatids and gets ligated to the 5′ end of every mRNA) for 2nd strand cDNA synthesis and sequencing. Two independent experiments were performed: run 1 on the Genome Analyzer IIx (Illumina) and run 2 on the HiSeq 2000 (Illumina). The Fastq files were read into the Spliced Leader ADDition (SLADD) program [29] and mapped onto the reference genome sequence of *T. brucei* TREU927 [2], using MAQ [30] with *n* = 3 and with a read length of ≥24. Multi-mapping reads were separated from single mappers by an alignment quality threshold of 30. Read counts were normalized according to library size and expressed as tags per million reads (TPM). Statistical analysis for differentially expressed genes was performed with the DESeq package in R [31]. DESeq uses a negative binomial distribution and a shrinkage factor for the distribution’s variance. Only mapped reads (raw counts) with a stringent quality score of *q* < 30 and data from both performed SLT runs were included.

### Whole genome sequencing

Whole genome sequencing of *T. b. rhodesiense* STIB900 was carried out on the Illumina HiSeq 2000 platform. Two times 12,243,924 paired-end reads of 76 b were mapped chromosome-wise to the reference genome *T. b. brucei* 927 (v5) using MIRA (v3.9.16) [32]. Gene models from the reference genome *T. b. brucei* 927 were transferred to the assembled STIB900 genome using rapid annotation transfer tool (RATT) [33] from the post assembly genome improvement toolkit (PAGIT) [34] package. Whole genome sequencing of *T. b. rhodesiense* STIB900-M and STIB900-P was carried out on the Genome Sequencer FLX Titanium by Roche/454. Two shotgun runs per line were performed. FASTQ format was extracted from .sff files using ‘SFF converter’ from Galaxy [35]. High-quality (HQ) reads were mapped to the assembled STIB900 genome, indexed with word length 13 and skip step 1, using the program SMALT (ftp.sanger.ac.uk/pub4/resources/software/smalt). Consensus sequence and variants relative to the assembled STIB900 genome were identified with ‘mpileup’ from SAMtools [36]. Ad hoc Perl scripts were used to compare nucleotide variants between the mapped reads of STIB900-M, STIB900-P, and the assembled STIB900 genome. For comparison also STIB900 reads generated on the Roche/454 platform were mapped to the assembled STIB900 genome. SNPs were called if they had a read depth of at least five high-quality bases (DP4 ≥ 5) and a read mapping quality of minimum 20 (mapq ≥20). All identified SNPs, indels, and gene deletions were inspected manually using Artemis [37]. The Roche/454 reads are accessible via the European Nucleotide Archive (http://www.ebi.ac.uk/ena) under accession number PRJEB12780.

### PCR amplification and Sanger sequencing

The PCR primers to amplify the 3.2 kb *AQP2*/*AQP3* tandem locus were AQP2/3_F (aagaaggctgaaactccacttg) and AQP2/3_R (tgcactcaaaaacaggaaaaga), annealing at 58 °C. *AT1*-*G430R* was amplified from genomic DNA of STIB900-P with primers AT1_F (gaaatccccgtcttttctcac) and AT1_R (atgtgctgagcctttttcctt), annealing at 56 °C. The PCR product was purified on silica-membrane columns (Nucleospin gel and PCR clean up, Macherey–Nagel) and digested with *Nru*I (New England Biolabs), run on a 1.5 % agarose gel and visualized with ethidium bromide. *UBP1* and *UBP1*-*R131L* were amplified from STIB900 and STIB900-M, respectively, with the primers UBP1_F (ccgctctagatctcaggttccactggcttc) and UBP1_R (ccgcggatcctcatttacgggcaggccgac).

### Plasmid construction, transfection and knock-out generation

The gene encoding the AT1-G430R mutant was amplified by PCR from genomic DNA of STIB900-P and the product was ligated into the expression vector pHD1336 [38] to give pHDK68. The plasmid was verified by Sanger sequencing (Source BioScience, Nottingham, UK) for the presence of the expected mutation and linearized with *Not*I prior to transfection into *T. b. brucei* clone B48, which lack the *AT1* gene and the high-affinity pentamidine transporter [16]. B48 parasites were washed in Human T Cell Solution for transfection with an Amaxa Nucleofector [39]. Transfectants were cloned by limiting dilution in standard HMI-11 medium [40] containing 5 µg/ml blasticidin for selection of the positive transfectants. Correct integration of the expression cassettes was tested by PCR.

*Trypanosoma brucei rhodesiense* 2T1 *aqp2/3* ^−*/*−^ cells were assembled using a I-*Sce*I meganuclease-based gene-conversion approach. Briefly, blasticidin deaminase (*BSD*) and neomycin-phosphotransferase (*NPT*) cassettes were used to replace the *AQP2/3* locus. Meganuclease cleavage of the *NPT* cassette was then used to trigger replacement with, and duplication of, the *BSD* cassette. *AT1* was disrupted in the resulting 2T1 *aqp2/3* ^*−/*−^ cells by replacing the first allele of the 1392 bp ORF with PCR-amplified *NPT* containing 100 bp overhangs identical to the UTRs of *AT1*, followed by selection with 5 µg/ml G418. The second allele was replaced with *PAC* plus 500 bp *AT1* UTR on either end, followed by selection with 0.1 µg/ml puromycin. Homozygous deletion of *AT1* was verified by PCR (Figure S1).

### Reverse genetics of UBP1

For overexpression of UBP1, the *UBP1* and *UBP1*-*R131L* PCR products were cloned into pRPai^GFPx^ [41] via *Xba*I and *BamH*I. *T. b. brucei* 2T1 cells [42] were transfected with *Asc*I-digested plasmids in Tb-BSF nucleofection buffer [43] using the Amaxa nucleofector (Lonza) with program Z-001. Transfectants were cloned by limiting dilution and selected with 2.5 µg/ml hygromycin. PCR and Sanger sequencing confirmed correct integration and sequence of the transgene.

To introduce the mutant *UBP1*-*R131L* in 2T1 cells in situ, a plasmid carrying the mutation and a blasticidin resistance gene (*BSD*) in the 5′ UTR of *UBP1*, used as a selection marker, was constructed (Supplementary Figure S3A). The synthetic DNA was obtained from GenScript (Piscataway Township, NJ, USA), integrated between the *Hin*dIII and *Bam*HI sites of cloning plasmid pUC57. DNA for transfection was prepared by PCR amplification of the insert using primers BLA_UBP1mut_F1 (ttgcattcgctcctttccct) and BLA_UBP1mut_R1 (ccttcagtagtttgttgagg) and subsequent purification as described above. 2T1 cells were transfected with an Amaxa Nucleofector using program Z-001 and clones were obtained as described above. Cells were selected with 5 and 10 µg/ml blasticidin. Correct homozygous integration was verified by PCR (Supplementary Figure S3B) and Sanger sequencing (Fig. 6b).

### SDS-PAGE and western blotting

Cells were lysed in NUPAGE^®^ LDS sample buffer (Life Technologies) and samples were loaded on precast 4–12 % Bis–Tris Gradient Gels (NuPAGE Novex^®^, Life Technologies) in MES running buffer and transferred to nitrocellulose membranes using the iBlot dry-blotting system (Novex^®^, Life Technologies). Membranes were blocked in 5 % milk in PBS/Tween-20 and incubated with primary antibodies in 5 % milk in TBS/Tween-20 overnight at 4 °C. Membranes were washed and incubated with peroxidase-conjugated secondary antibodies in 5 % milk PBS/Tween-20 for 2 h at room temperature. Blots were developed using the ECL Western Blotting Substrate (Pierce) using a ChemiDoc™ MP Gel Imaging System (Biorad). Primary Antibodies used: rabbit anti-GFP (Abcam, Ab290), mouse anti-BiP (kind gift of Prof A. Schneider). Secondary Antibodies used: goat anti-rabbit (SouthernBiotech: 4050-05), polyclonal rabbit anti-mouse HRP (Dako, Baar, Switzerland).

## Results

### Phenotypic profiling of high-level melarsoprol–pentamidine cross-resistance

Before venturing into genomics we performed an in-depth phenotypic profiling of the parental *T. b. rhodesiense* STIB900 and its two drug-resistant derivatives STIB900-M and STIB900-P. Drug sensitivities were quantified as 50 % inhibitory concentrations (IC_50_) towards the five clinical trypanocides (pentamidine, suramin, melarsoprol, eflornithine, and nifurtimox), two clinical candidates (fexinidazole and the diamidine DB75), and selected experimental compounds (Table 1). STIB900-M and STIB900-P exhibited similar resistance profiles, namely strong MPXR with cross-resistance to other diamidines (i.e., diminazene aceturate and DB75) and adenosine analogs [cordycepin (3′-deoxyadenosine), tubercidin (7-deazaadenosine), vidarabine (adenosine arabinoside)]. The melarsoprol-selected line generally had higher resistance factors than the pentamidine-selected line to the adenosine analogs and other typical AT1 substrates [23] such as DB75 and melarsoprol itself, though not all the differences were statistically significant (*p* < 0.004 in Anova one-way analysis of variance followed by Tukey’s multiple comparison test; Table 1). On the other hand, STIB900-P was cross-resistant to the adenine analog aminopurinol (not a AT1 substrate [44]) whereas STIB900-M was not (Table 1). Neither line was resistant (*p* < 0.004 in Anova plus Tukey’s test) to suramin, nifurtimox, fexinidazole, or phenylarsine oxide, a hydrophobic arsenical that diffuses across the plasma membrane [13]. Surprisingly, both STIB900-M and STIB900-P were significantly hypersensitive to eflornithine (difluoromethylornithine, DFMO). This unexpected result is consistent with the observation that eflornithine resistance caused by loss of the amino acid transporter TbAAT6 was accompanied by hypersensitivity to pentamidine [45]. *TbAAT6* was not overexpressed in the resistant lines (see below). The in vitro population doubling times without drug were 9.4 ± 0.3 h and did not significantly differ between the tree lines (*p* > 0.05 in Anova one-way analysis of variance followed by Tukey’s multiple comparison test).

**Table 1:** In vitro drug sensitivity profiles

	IC_50_ ± standard deviation [nM]			*p* value			Resistance factor	
	STIB900	STIB900-P	STIB900-M	STIB900 vs. 900-P	STIB900 vs. 900-M	STIB900-P vs. 900-M	STIB900-P to 900	STIB900-M to 900
Melarsoprol	6.0 ± 3.4	84 ± 52	170 ± 63	0.040	<0.0001	0.026	14	28
Pentamidine	2.8 ± 0.8	130 ± 69	210 ± 93	0.033	0.0008	0.17	47	76
Diminazene	3.8 ± 1.5	18 ± 5.4	25 ± 10	0.012	0.0003	0.25	4.7	6.5
DB75	3.7 ± 0.9	22 ± 8	64 ± 17	0.046	<0.0001	<0.0001	5.9	17
Suramin	135 ± 62	76 ± 32	125 ± 25	0.11	0.92	0.21	0.6	0.9
Nifurtimox	1100 ± 550	1100 ± 460	1500 ± 620	0.99	0.51	0.55	1.0	1.4
Fexinidazole	3200 ± 1100	2500 ± 980	6100 ± 2500	0.80	0.041	0.013	0.8	1.9
Eflornithine	5200 ± 1400	1300 ± 640	2700 ± 480	<0.0001	0.0033	0.094	0.3	0.5
Cordycepin	0.46 ± 0.1	2.2 ± 0.54	7.0 ± 1.5	0.026	<0.0001	<0.0001	4.7	15
Vidarabine	265 ± 108	400 ± 52	650 ± 190	0.24	0.0006	0.0186	1.5	2.5
Tubercidin	26 ± 6	100 ± 19	4300 ± 1000	0.97	<0.0001	<0.0001	3.8	163
Aminopurinol	1500 ± 190	5400 ± 1100	2000 ± 630	<0.0001	0.58	<0.0001	3.6	1.3
Phenylarsine	0.52 ± 0.13	0.65 ± 0.11	0.75 ± 0.08	0.26	0.028	0.45	1.3	1.4

Mean IC_50_ values, *p* values of Anova one-way analysis of variance followed by Tukey’s multiple comparison test, and resistance factors. Taking into account the 13 independent tests of the different drugs, the Bonferroni-corrected threshold for significance is *p* < 0.00385

### Transcriptomic profiling indicates loss of expression of transporter genes

**Fig. 1:**
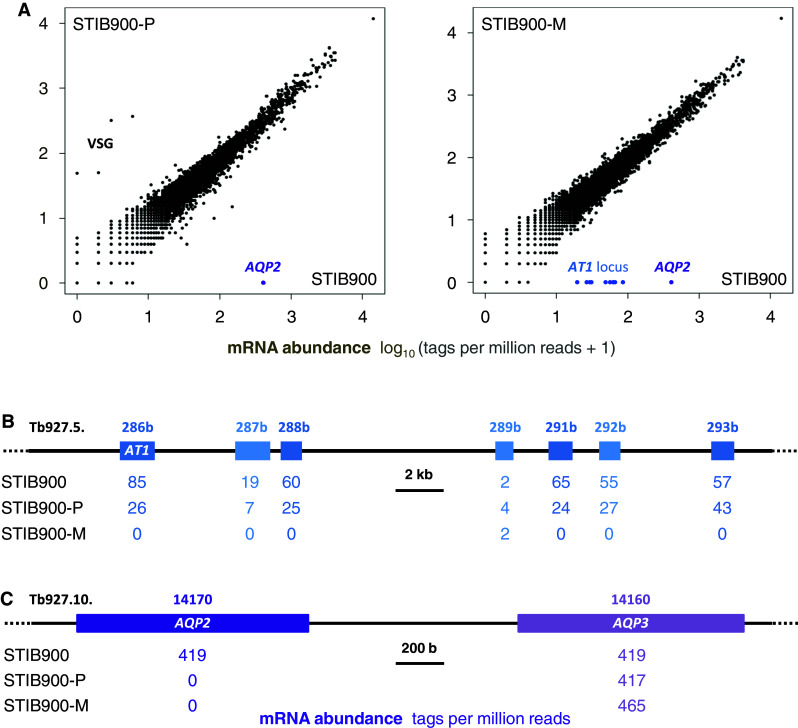
Comparative transcriptomics. **a** Scatter plots of normalized read counts from RNA sequencing data using the spliced leader trapping protocol. The increment of one allowed logarithmic representation also for genes that had zero sequence tags. Genes that are not expressed in the *resistant lines* are indicated. Note the *VSG* switch in STIB900-P. **b** View of the *AT1* locus with read counts per gene. **c** View of the *AQP2/AQP3* tandem locus with read counts per gene

**Fig. 2:**
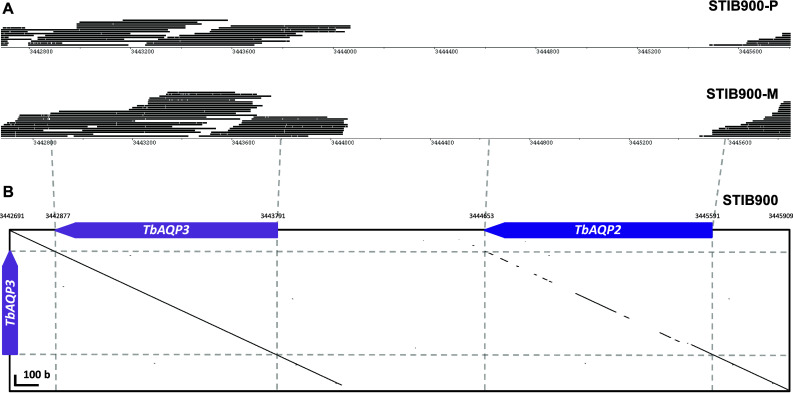
Loss of *AQP2* from the resistant lines. **a** Roche-454 sequencing reads generated from genomic DNA of STIB900-P and STIB900-M mapped to the reference sequence of STIB900 as visualized with BamView [59]. The gene *AQP2* is missing in both STIB900-P and STIB900-M. **b** The tandem nature of the *AQP2/AQP3* locus on chromosome 10 as illustrated by a *dot plot* of the STIB900 reference sequence from (**a**) on the *x axis* vs. the sequence of *AQP3* on the *y axis* (made with GEPARD [60] and a word length of 10). The genes *AQP2* and *AQP3* are highly similar (82 % global identity) as depicted by the diagonal on the *right*

Quantitative transcriptomics served as a first tool to investigate the strong MPXR phenotype of *T. b. rhodesiense* STIB900-P and STIB900-M at the molecular level. For this purpose we used the ‘spliced leader trapping’ (SLT) adaptation of Illumina RNA-Seq [29], exploiting the fact that all trypanosomal mature mRNAs carry the same 39 nt leader sequence spliced in-trans to their 5′ end, a peculiarity of trypanosomatids [46]. SLT is optimally suited to quantify steady-state mRNA levels by counting the number of reads per transcript because all the reads stem from the transcription start sites. Figure 1 shows one of two independent experiments; all data are included in the supplementary Excel file *Graf_S1.xlsx*. Overall, there was very little variance between the drug-resistant lines and their sensitive parent (Fig. 1a). Four genes were overexpressed in STIB900-P compared to STIB900 and STIB900-M, all of which are neighboring genes on chromosome 6 encoding for VSGs. This confirms that antigenic variation also takes place in vitro, but new variants rarely become fixed in the population because of continuous dilution of the growing cultures. Apart from these *VSG*, no genes were significantly overexpressed in the resistant *T. b. rhodesiense* lines—including TbMRPA, an export pump that confers resistance to melarsoprol when overexpressed ectopically [47, 48]. The adenosine transporter *AT1* and six adjacent genes on the telomere of chromosome 5 [49] were not expressed above detection limit in STIB900-M (Fig. 1b). This is in agreement with the reported absence of *AT1* in STIB900-M [24] and is indicative of a larger deletion at the *AT1* locus. The aquaglyceroporin *AQP2* appeared not to be expressed above detection limit in either resistant line (Fig. 1c). However, the automated mapping of the reads [29] was ambiguous because of the high degree of similarity between *AQP2* and *AQP3* at their 5′ ends (Fig. 2). The short Illumina reads were manually mapped to *AQP3* since the deletion of *AQP2* was confirmed by the longer 454 reads (Fig. 2). Apart from *AT1* plus adjacent genes and *AQP2*, no genes were significantly under expressed in the resistant lines.

### A reference genome sequence of the *T. b. rhodesiense* drug-sensitive parent

Before exploring the mutations underlying the strong MPXR phenotype of STIB900-P and STIB900-M by comparative genomics, we had to generate a good-quality draft genome of the susceptible parent *T. b. rhodesiense* STIB900. Genomic DNA was isolated from bloodstream-form trypanosomes grown in mice. The obtained gDNA was verified to be free of mouse DNA by PCR with primers for mouse GAPDH. Paired-end Illumina reads generated on the HiSeq platform were mapped to the core chromosomes of *T. b. brucei* TREU927 with an average coverage of 53 fold. The vast majority of gene models (9692 of 9722) were transferred from *T. b. brucei* TREU927 to the assembled *T. b. rhodesiense* STIB900 genome, identifying a total of 112,565 high-quality single-nucleotide polymorphisms (SNP) between these two genomes. In protein-coding regions there were 46,453 SNPs, of which 19,575 non-synonymous. As expected, the assembled *T. b. rhodesiense* STIB900 genome contained the *SRA* gene (serum resistance-associated; Tb927.9.17380), whose product neutralizes ApoL1, the trypanolytic factor of human serum that protects humans from infection by *T. b. brucei*. The genome reference strain *T. b. brucei* TREU927 contains a dysfunctional SRA ortholog [50, 51].

### Comparative genomics confirms the loss of transporter genes

The assembled genome sequence of *T. b. rhodesiense* STIB900 was used as a reference to identify mutations in the resistant derivatives STIB900-P and STIB900-M. Genomic DNA was isolated from bloodstream-form trypanosomes harvested from infected mice. The known absence of *AT1* from STIB900-M was used for diagnostic PCR of the purified gDNA to verify that there had been no contaminations. Roche-454 sequencing libraries were generated for all three lines, obtaining ~1.5 million high-quality reads for each genome, corresponding to a 20-fold coverage (Table S1). The Roche/454 reads from STIB900-M and STIB900-P were mapped to the assembled STIB900 genome with an overall coverage of 83 % (DP4 ≥5). SNPs and indels were identified with SAMtools and with self-developed Perl scripts. All identified SNPs, indels, and gene deletions were inspected manually using Artemis [37]. Overall, there were remarkably few mutations in STIB900-M and STIB900-P relative to their parent STIB900 (Table 2). Only one coding point mutation was found in both resistant lines (in the gene *UBP1*, see below). Both lines carried a deletion of about 1.8 kb at the *AQP2/AQP3* locus (Fig. 2a), causing loss of the aquaglyceroporin *AQP2* but not of *AQP3*. The deletion was confirmed by PCR on genomic DNA followed by sequencing of the products (not shown). *T. b. rhodesiense* STIB900-M had not only lost the gene *AT1* (Tb927.5.286b), as previously published [24], but a whole region of over 25 kb encompassing *AT1* and the adjacent genes Tb927.5.288b (ribulose-phosphate 3-epimerase, putative), Tb927.5.289b (hypothetical protein), Tb927.5.291b (variant surface glycoprotein-related), and Tb927.5.292b (hypothetical protein).

**Table 2:** SNP statistics

	STIB900-M vs. STIB900			STIB900-P vs. STIB900		
	Overall	CDS	NS	Overall	CDS	NS
Chr1	0	0	0	2	0	0
Chr2	0	0	0	0	0	0
Chr3	0	0	0	1	0	0
Chr4	0	0	0	0	0	0
Chr5	2	0	0	1	Tb927.5.286b	G430R
Chr6	1	0	0	0	0	0
Chr7	0	0	0	1	0	0
Chr8	1	0	0	0	0	0
Chr9	6	0	0	5	Tb927.9.8310*	D31G
Chr10	0	0	0	0	0	0
Chr11	2	Tb927.11.500	R131L	4	Tb927.11.500	R131L
Total	12	1	1	14	3	3

Single-nucleotide polymorphisms between the drug-resistant *T. b. rhodesiense* lines and their parent STIB900 (*CDS* coding sequence, *NS* non-synonymous)

* This gene is no longer part of TriTrypDB

### A point mutation that renders TbAT1 non-functional is heterozygous in STIB900-P

**Fig. 3:**
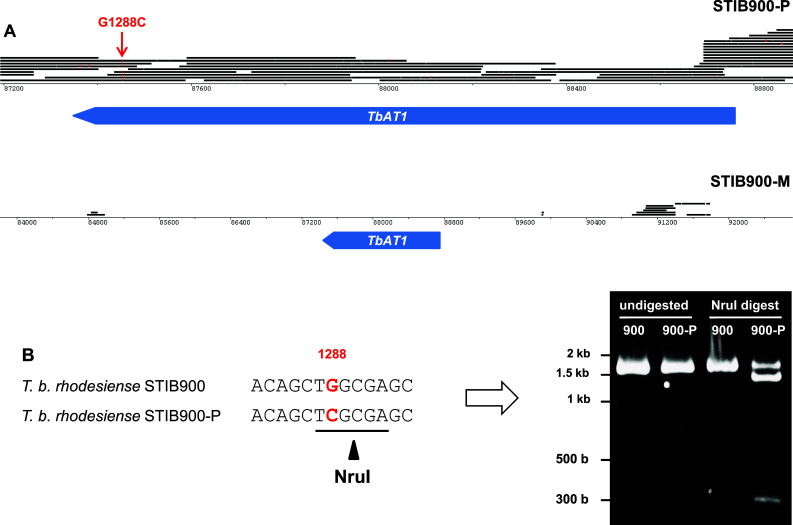
Loss or mutation of *AT1* in the *resistant lines*. **a** Genomic 454 reads of STIB900-P and STIB900-M mapped to STIB900 visualized for the *AT1* locus on chromosome 5 using BamView [59] (smaller scale for STIB900-M). There is a deletion of *AT1* in STIB900-M and a coding point mutation in STIB900-P (*red*). **b** *AT1* PCR products (1636 bp) were amplified from genomic DNA of STIB900 and STIB900-P, and digested with the endonuclease *Nru*I. G1288C mutant alleles are cut to fragments of 1339 and 257 bp. STIB900-P appears to be heterozygous for the mutation

**Fig. 4:**
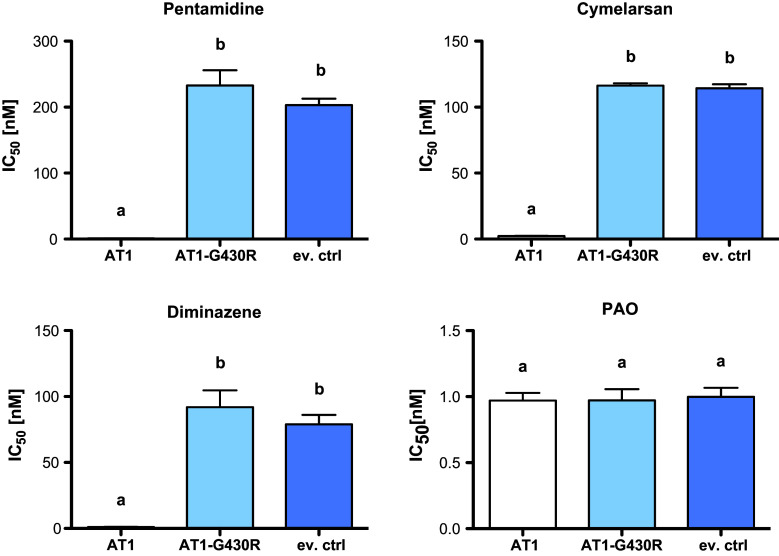
Functional characterization of G430R mutant *AT1*. The genes *AT1*, *AT1*-*G430R*, and an empty vector control (ev. ctrl) were expressed in the *T. b. brucei* B48 mutant [16]. Transfectant clones were tested in vitro for their sensitivity to the known AT1 substrates pentamidine, cymelarsan, and diminazene. Phenylarsine oxide (PAO) was included as a negative control that is not a AT1 substrate. The IC_50_ values are from 5 to 8 independent experiments; *error bars* are standard errors of the mean. The *small letters* indicate significance groups (*p* < 0.05) as determined by Anova one-way analysis of variance followed by Tukey’s multiple comparison test

*Trypanosoma brucei rhodesiense* STIB900-P still possessed *AT1* but the gene contained a non-synonymous substitution, G1288C, resulting in the mutation of glycine^430^ to arginine. The identified point mutation was confirmed with Sanger sequencing and by restriction digest of the *AT1* PCR product, since the mutation generated an endonuclease *Nru*I site (tcgcga; Fig. 3). The facts that the *AT1* PCR products from genomic DNA of STIB900-P (1) were not digested completely (Fig. 3), and (2) after cloning and sequencing did not all contain the C at position 1288 (not shown), indicate that STIB900-P is heterozygous for the mutation G1288C. The AT1-G430R mutant was functionally characterized in *T. b. brucei* B48, a mutant which lacks high-affinity transport of melarsoprol and pentamidine [16, 52]. B48 bloodstream-form cells were transfected with wildtype *AT1*, G430R mutant *AT1*, and empty vector as a control. Expression of wildtype *AT1* strongly sensitized the B48 transfectants to melarsoprol, pentamidine, and diminazene aceturate (Berenil^®^), whereas G430R mutant *AT1* did not (Fig. 4). This demonstrates that the point mutation renders AT1 non-functional with respect to drug transport.

### Concomitant deletion of AQP2 and AT1 does not phenocopy the high-level MPXR

**Fig. 5:**
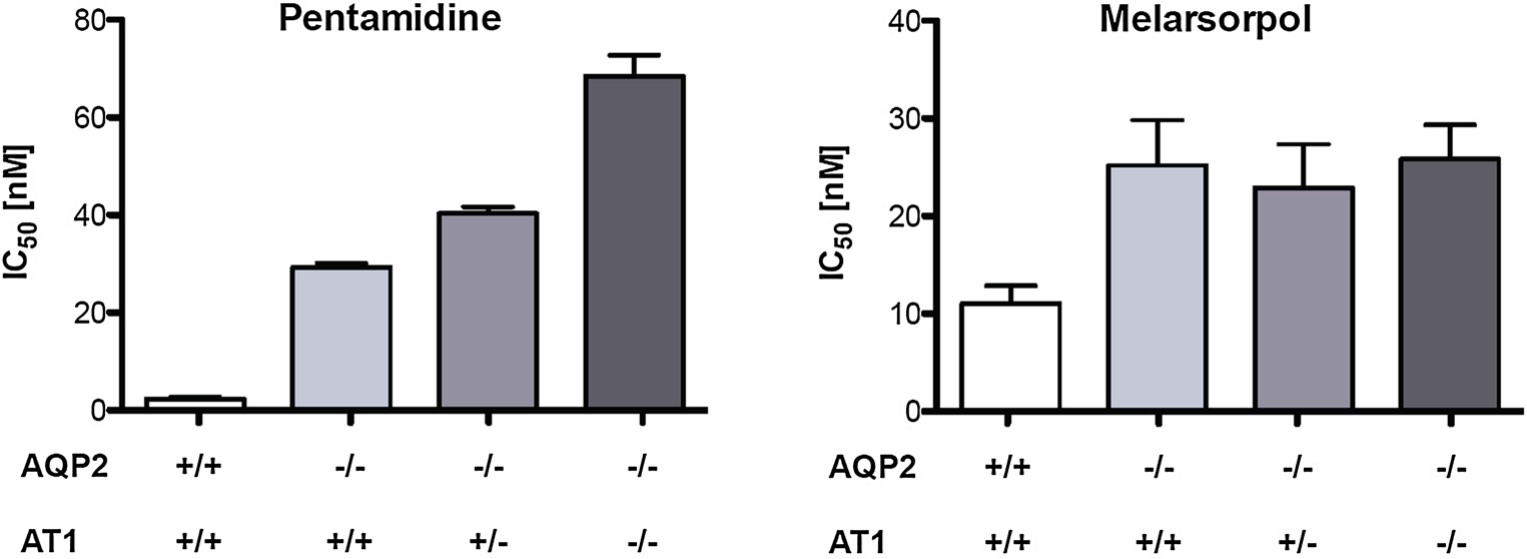
*AT1/AQP2* double knock-out in *T. brucei*. In vitro drug sensitivities were determined for pentamidine and melarsoprol on parental 2T1 cells, 2T1-*aqp2* ^−*/*−^, 2T1-*aqp2* ^−*/*−^-*AT1* ^+*/*−^, and 2T1-*aqp2* ^−*/*−^-*at1* ^−*/*−^. *Bars* are the average IC_50_ of at least four independent experiments, each performed in triplicate. *Error bars* indicate the standard error of the mean

The contributions to MPXR of either *AQP2* or *AT1* alone have been extensively studied [11, 53]. To try and phenocopy *T. b. rhodesiense* STIB900-P and STIB900-M, respectively, we have generated heterozygous and homozygous *T. b. brucei* *at1* null lines (Supplementary Figure S1) in a *aqp2/aqp3* null background [17]. The four lines 2T1 (parental), 2T1-*aqp2*/*aqp3* ^−/−^, 2T1-*aqp2*/*aqp3* ^−/−^-*at1* ^+/−^, and 2T1-*aqp2*/*aqp3* ^−/−^-*at1* ^−/−^ were characterized regarding their growth and drug sensitivities. The four lines grew equally well in vitro with population doubling times of around 7.0 ± 0.5 h (*p* > 0.05 in Anova one-way analysis of variance followed by Tukey’s multiple comparison test), indicating that simultaneous loss of *AT1*, *AQP2*, and *AQP3* does not bear a fitness cost in vitro. In agreement with previous findings [17], the IC_50_ of pentamidine increased by about 15 fold in the absence of *AQP2*. Heterozygous deletion of *AT1* further increased the resistance factor to 20, homozygous deletion to 35 (Fig. 5). The susceptibilities of the four strains to melarsoprol did not follow such a pronounced pattern, with moderated resistance factors of 2–2.5 (Fig. 5). Clearly, the MPXR resistance factors obtained through simultaneous knock-out of *AT1* and *AQP2* were below those of *T. b. rhodesiense* STIB900-M and STIB900-P (Table 1), indicating that further mutations must contribute to the high-level MPXR phenotype observed in those lines.

### Testing of the RNA-binding protein UBP1 as a new candidate resistance gene

**Fig. 6:**
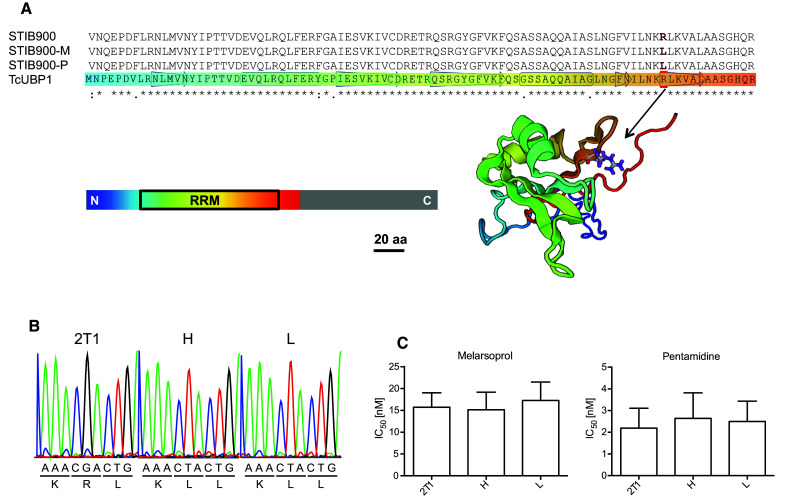
In situ mutation of *UBP1* in *T. b. brucei*. **a** Alignment of the predicted RNA recognition motif (RRM) of UBP1 from STIB900, STIB900-M, and STIB900-P alongside *Trypanosoma cruzi* UBP1. The mutation of the conserved Arg^131^ to Leu is highlighted in the alignment and Arg^131^ is indicated with an *arrow* in the published structure of TcUBP1. The full length TcUBP1 is 224 aa but a truncated version (1–139) including the RRM (35–126) was used to determine the structure [54]. Swiss model [61] was used to visualize the structure. **b** Sanger sequencing electropherograms of *UBP1* amplified from genomic DNA of parental 2T1 cells and two transgenic clones H and L, showing the nucleotides that encode amino acids 130–132. **c** In vitro drug sensitivities (IC_50_ ± standard deviation; *n* = 12) for melarsoprol and pentamidine determined with the Alamar blue assay for parental 2T1 cells and the two transgenic clones H and L. There was no significant difference in drug sensitivity between the *three lines* (Anova one-way analysis of variance followed by Tukey’s multiple comparison test)

In view of the very small number of non-synonymous SNPs (Table 2), the finding that the two independently selected lines, STIB900-P and STIB900-M, were homozygous for the same point mutation, Arg^131^ to Leu, in the uridine-rich-binding protein 1 (UBP1, Tb927.11.500; Table 2) was of particular interest. UBP1 is an RNA-binding protein with a single, conserved RNA recognition motif (RRM, Fig. 6a), whose structure has been determined for its ortholog in *Trypanosoma cruzi* [54]. Arg^131^ of TbUBP1 corresponds to Arg^113^ of TcUBP1 in *T. cruzi* and lies within the ß4–ß5 hairpin of the RRM (Fig. 6a) that is involved in RNA binding [54]. To test the impact of the mutation R131L on TbUBP1 function, we first generated two stable *T. b. brucei* 2T1 cell lines overexpressing either GFP-UBP1-wt or GFP-UBP1-R131L at a single specific locus within the rRNA spacer in a tetracycline (Tet)-inducible manner [42]. Both lines still possessed the endogenous *UBP1* alleles. Overexpression of GFP-UBP1-wt for longer than 24 h caused a strong growth defect (Supplementary Figure S2A), which is in agreement with previous findings [55]. Interestingly, overexpression of GFP-UBP1-R131L did not cause a growth phenotype (Figure S2A). However, the GFP-UBP1-R131L overexpressing trypanosomes were slightly but significantly hypersensitive to pentamidine as compared to non-induced cells (IC_50_ of 1.2 nM vs. 2.7 nM; Figure S2B). We do not have an explanation for this counter-intuitive result—which has to be interpreted with caution due to the lack of a control since overexpression of wildtype UBP1 was toxic to the trypanosomes. Attempting to test the role of the identified mutation in a more physiological context, we introduced the mutant gene *UBP1*-*R131L* in *T. b. brucei* 2T1 cells in situ. A blasticidin resistance gene (*BSD*) was added to the 5′ UTR of the *UBP1* gene together with the mutation encoding for Leu^131^ (Supplementary Figure S3A). Antibiotic pressure allowed selection of two homozygous clones (designated H and L) without the need for a second round of transfection (Fig. 6b, Supplementary Figure S3B). However, neither of the two clones exhibited a significantly reduced sensitivity to melarsoprol or pentamidine as compared to parental 2T1 cells (Fig. 6c).

## Discussion

Cross-resistance of African trypanosomes to melarsoprol and pentamidine (MPXR) is a well-known phenomenon [11]. Here we perform in-depth phenotypic and genotypic profiling of two lab-derived *T. b. rhodesiense* mutants with most pronounced MPXR phenotypes: STIB900-P, selected with pentamidine, and STIB900-M, selected with melarsoprol. The two independently selected lines exhibited similar—but not identical (Table 1)—resistance profiles, extending MPXR to other diamidines and adenosine analogs, but not to suramin or nifurtimox.

Very few mutations had become fixed in the drug-selected lines in the 2 years’ course of selection (Table 2), and very few genes were differentially expressed in the resistant lines compared to the sensitive parent (Fig. 1). The only striking difference was the complete absence of RNA-Seq reads for *AQP2* in STIB900-M and STIB900-P, and for *AT1* and neighboring genes in STIB900-M. Both STIB900-M and STIB900-P carried a deletion of the *AQP2* locus (Fig. 2). This ‘natural knock-out’ of *AQP2* was probably due to homologous recombination with the neighboring, highly similar gene *AQP3* (Fig. 2), accompanied by loss of genetic material. Cases of truncation or chimerization of *AQP* genes in drug-resistant *T. brucei* had been reported previously [17, 20, 21, 56]. *T. b. rhodesiense* STIB900-M also carried a large deletion on chromosome five encompassing *AT1* and six adjacent genes (Fig. 3), a gene loss possibly facilitated by their telomeric location. STIB900-P had a non-synonymous point mutation in *AT1* changing Gly^430^ to Arg (Fig. 3). Expression of wildtype and G430R-mutant *AT1* in a *T. b. brucei* loss of transport mutant demonstrated that the AT1-G430R did not transport melarsoprol or diamidines (Fig. 4). The finding that STIB900-P is heterozygous for the mutation might explain its milder MPXR phenotype than STIB900-M (Table 1). These results are the best evidence to date that the MPXR models developed in the non-human infective trypanosome *T. b. brucei* hold true in *T. b. rhodesiense*, the causative agent of sleeping sickness in East Africa, and that selection for high-level resistance to melarsoprol and pentamidine can lead to loss of both known drug transporters.

To investigate the combined contribution of *AQP2* and *AT1* to drug sensitivity, we constructed *T. b. brucei* hetero- and homozygous *at1* null mutants in a *aqp2/aqp3* null background. The effects of the mutations were additive regarding pentamidine sensitivity, with a maximal resistance factor of 35 for the double null mutant of *at1* and *aqp2* (Fig. 5). The phenotypes with respect to melarsoprol were less pronounced, as the loss of *AT1* did not further increase the melarsoprol resistance of the *aqp2* null mutant (Fig. 5). The conclusion from these experiments is that concomitant deletion of *AQP2* and *AT1* does not completely phenocopy the strong MPXR phenotypes of *T. b. rhodesiense* STIB900-M and STIB900-P, demonstrating that further genes must be involved. Only one additional gene was affected by a mutation in both resistant lines: the RNA-binding protein *UBP1*.

*Trypanosoma brucei rhodesiense* STIB900-M and STIB900-P both carried the mutation R131L in UBP1, which was absent in the parent and must have been acquired by the resistant lines independently. The precise function of UBP1 is unknown, but it has been implicated in regulation of mRNA levels [55]. Based on the alignment to TcUBP1 from *T. cruzi*, Arg^131^ of TbUBP1 is predicted to be critical for RNA binding (Fig. 6a), suggesting that Leu^131^ may impair TbUBP1 function. Complete loss of UBP1 function may be lethal, as indicated by RNAi-mediated knock-down of *UBP1* and *UBP2* [55]. TcUBP1 is a cytoplasmic protein in epimastigotes of *T. cruzi* [57]. Intriguingly, TcUBP1 was shown to accumulate in the nucleus when the trypanosomes were under arsenite stress, and mutations affecting RNA binding prevented nuclear accumulation of TcUBP1 [57]. *TbUBP2*, which shares 73 % global identity with *TbUBP1*, had appeared as a secondary hit in a genome-wide RNAi screen for pentamidine resistance in *T. brucei* [58]. While such previous findings may support a possible role of UBP1 in drug resistance, our reverse genetic approaches did not. Non-physiological overexpression of UBP-Leu^131^-GFP in *T. b. brucei* bloodstream forms even caused a slight hypersensitivity to pentamidine (Figure S2B). However, this needs to be interpreted cautiously since, in agreement with a previous report [55], overexpression of the ‘wildtype’ UBP1-GFP fusion protein was lethal (Figure S2A). More physiological in situ expression of the mutant *UBP1* in *T. b. brucei* did not affect the sensitivity to melarsoprol or pentamidine (Fig. 6c). In summary, if the mutation Arg^131^ to Leu in UBP1 contributes to drug resistance at all, then it does so only in the context of the described loss of AQP2 and/or AT1.

### Electronic supplementary material

The following additional data are available with the online version of this paper. Table S1 is a Excel file (Graf_TableS1.xlsx) of the normalized read counts as tags per million reads (TPM) for STIB900, STIB900-P, and STIB900-M for run 1 and run 2 from the RNA-Seq data. Table S2 is a Word file (Graf_TableS2.pdf) of the mapping statistics of Roche 454 reads of STIB900, STIB900-P, and STIB900-M to the reference genome *T. b. brucei* TREU 927. Figure S1 (Graf_FigureS1.pdf) is the PCR verification of *AT1* deletion in *T. b. brucei aqp2/aqp3* ^*-/-*^ cells. Figure S2 shows the results from overexpression of mutant *UBP1* in *T. brucei* (Graf_FigureS2.pdf) and Figure S3 illustrates the constructs made for *in situ* expression of mutant *UBP1* in *T. brucei* (Graf_FigureS3.pdf).

Supplementary material 1 (PDF 114 kb)

Supplementary material 2 (PDF 173 kb)

Supplementary material 3 (PDF 193 kb)

Supplementary material 4 (XLSX 579 kb)

Supplementary material 5 (PDF 46 kb)

## Abbreviations

MPXR: Melarsoprol–pentamidine cross-resistance
SLT: Spliced leader trapping
AQP2: Aquaglyceroporin 2
AT1: Adenosine transporter 1
Tet: Tetracycline
UBP1: Uridine-rich binding protein 1
VSG: Variant surface glycoprotein
