# Supplementary material for: Comparative genomics of drug resistance in Trypanosoma brucei rhodesiense
Source: Cell Mol Life Sci. 2016 Mar 14;73(17):3387–400. doi: 10.1007/s00018-016-2173-6 (PMC4967103; doi:10.1007/s00018-016-2173-6)
Supplement: Supplementary file 1 — Supplementary material 1 (PDF 114 kb) [file 18_2016_2173_MOESM1_ESM.pdf]

Graf, Ludin et al. 2016

Comparative genomics of drug resistance in *Trypanosoma brucei rhodesiense*

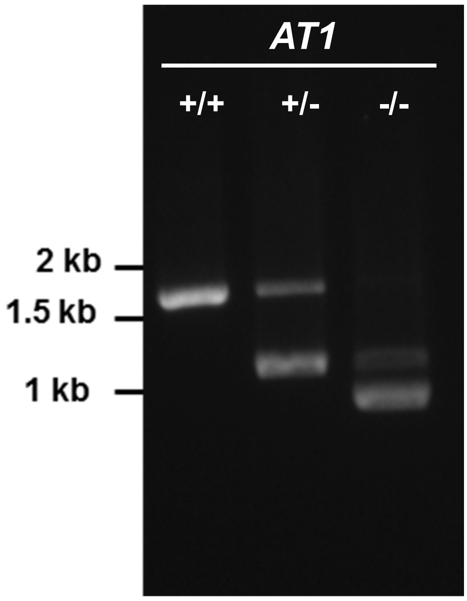

Supplementary Figure 1: Genetic knock-out of *AT1* in *aqp2/aqp3* double-null *Trypanosoma brucei brucei*

1% agarose gel of PCR amplified *AT1* gene from *T. b. brucei* 2T1 *aqp2/aqp3*$^{-/-}$ parental cells (+/+) and heterozygous (+/-) or homo-zygous (-/-) deletion of *AT1*. Primers were AT1_f (gaaatccccgtcttttctcac) and AT1_r (atgtgctgagcctttttcctt) at 56 °C annealing. The expected size for *AT1* is 1636 b; neomycin phosphotransferase (*NPT*) was used to replace the first allele (1051 b), puromycin-N-acetyltransferase (*PAC*) for the second allele (862 b).
