# Supplementary material for: Comparative genomics of drug resistance in Trypanosoma brucei rhodesiense
Source: Cell Mol Life Sci. 2016 Mar 14;73(17):3387–400. doi: 10.1007/s00018-016-2173-6 (PMC4967103; doi:10.1007/s00018-016-2173-6)
Supplement: Supplementary file 2 — Supplementary material 2 (PDF 173 kb) [file 18_2016_2173_MOESM2_ESM.pdf]

Graf, Ludin et al. 2016

Comparative genomics of drug resistance in *Trypanosoma brucei rhodesiense*

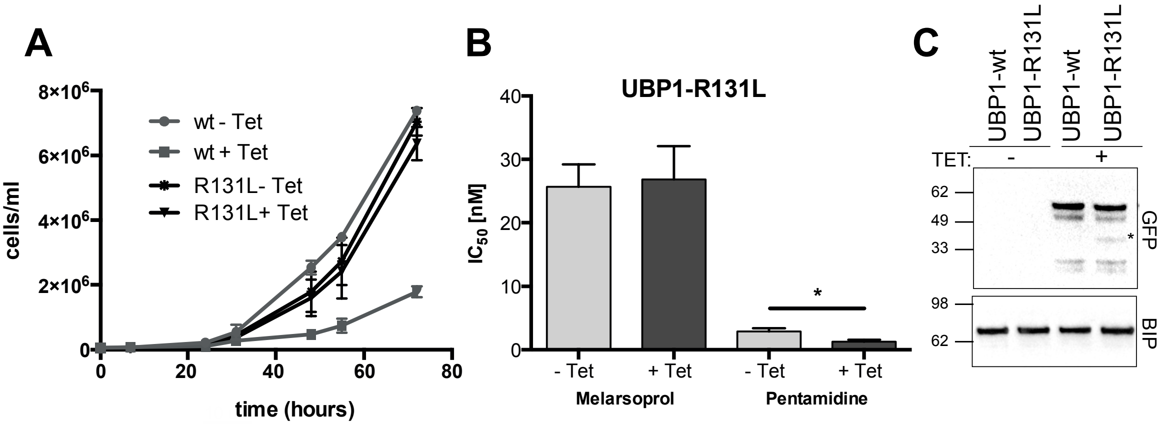

Supplementary Figure 2: **Overexpression of wild-type and mutant *UBP1* in *Trypanosoma brucei brucei***

A) Growth assay for GFP-*UBP1*-wt and GFP-*UBP1*-R131L transfected cells. Tetracycline induction of transgene expression was done at 1 µg/ml 24 hours before the first measurement. Error bars are standard deviations (n=3). Overexpression of GFP-*UBP1*-wt is toxic to trypanosomes (grey squares).

B) *In vitro* drug sensitivities (IC_{50}) for melarsoprol and pentamidine determined with the Alamar blue assay for GFP-*UBP1*-R131L overexpressing cells. Error bars are standard errors of the mean (n=10). The asterisk denotes significant difference ($p<0.05$) as determined by two-tailed t-test (GraphPad Prism 5.0.).

C) Western blot with anti-GFP antibody showing inducible expression of GFP-*UBP1*-wt and GFP-*UBP1*-R131L 24 hours after tetracycline addition. After 24 hours of overexpression an additional band of about 40 kDa reproducibly appeared in the Western blots of GFP-UBP1-R131L, but not GFP-UBP1-wt, which might indicate partial degradation of the mutant protein.
