# Supplementary material for: Comparative genomics of drug resistance in Trypanosoma brucei rhodesiense
Source: Cell Mol Life Sci. 2016 Mar 14;73(17):3387–400. doi: 10.1007/s00018-016-2173-6 (PMC4967103; doi:10.1007/s00018-016-2173-6)
Supplement: Supplementary file 3 — Supplementary material 3 (PDF 193 kb) [file 18_2016_2173_MOESM3_ESM.pdf]

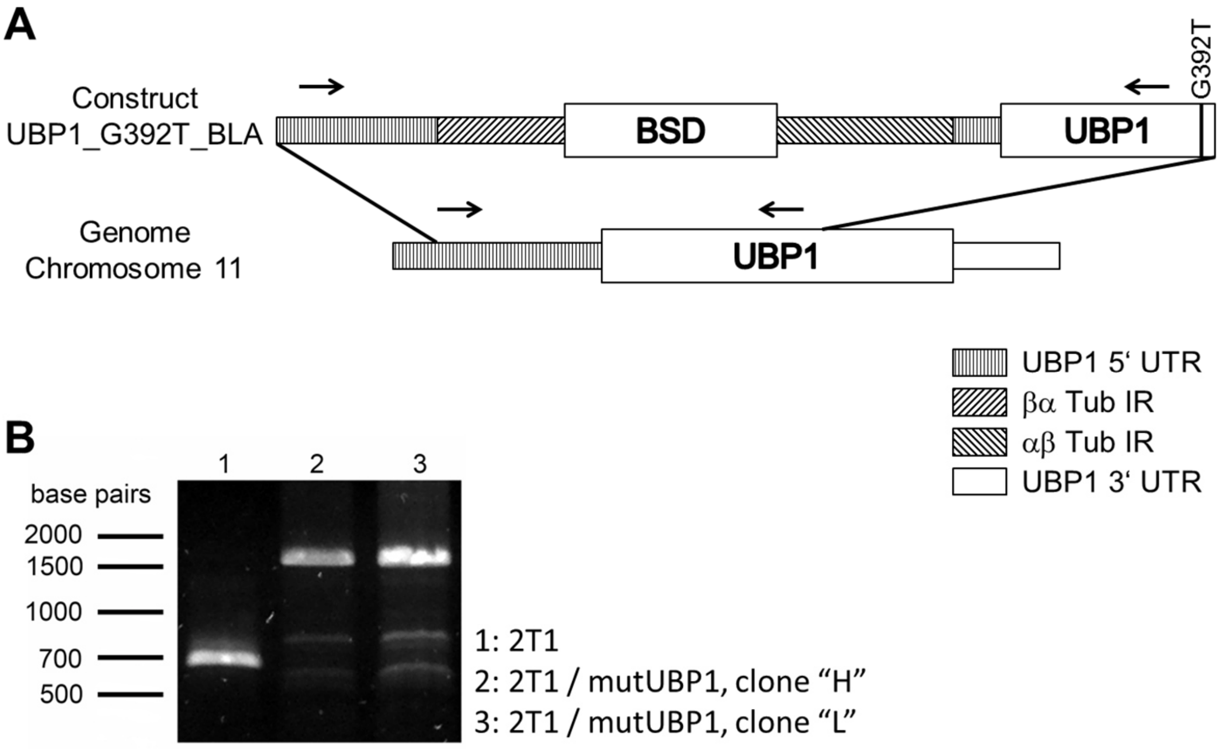

Supplementary Figure 3: *In situ* expression of mutant *UBP1* in *Trypanosoma brucei brucei*

A) Structure of the *UBP1* locus (bottom) and the construct used for *in situ* introduction of the mutation G392T (BSD, blasticidin-S deaminase, UTR, untranslated region; Tub IR, tubulin intergenic region). Primer binding sites used for PCR (B) are indicated with arrows.

B) 1% agarose gel of PCR products from parental 2T1 cells (1) and two transgenic clones (2, 3). The expected size is 697 b for 'wild-type' *UBP1* and 1678 b for the construct with the mutant *UBP1*. Both transgenic clones are homozygous for the mutant *UBP1* gene as based on the absence of the 697 b band. Primers used were UBP1-2_IR_fwd (tgcattcgctcctttccctt) and UBP1_rev (gacgagtctcccgatcacac).
