# Supplementary material for: Comparative genomics of drug resistance in Trypanosoma brucei rhodesiense
Source: Cell Mol Life Sci. 2016 Mar 14;73(17):3387–400. doi: 10.1007/s00018-016-2173-6 (PMC4967103; doi:10.1007/s00018-016-2173-6)
Supplement: Supplementary file 5 — Supplementary material 5 (PDF 46 kb) [file 18_2016_2173_MOESM5_ESM.pdf]

Graf, Ludin et al. 2015

Comparative genomics of drug resistance of the sleeping sickness parasite *Trypanosoma brucei rhodesiense*

	STIB900	STIB900-M	STIB900-P
High-quality (HQ) reads	1,418,416	1,513,957	1,482,415
Average read length	330 b	362 b	371 b
Calculated coverage	18-fold	21-fold	21-fold
Mapped reads (all mapping quality)	1,212,557	1,205,716	1,201,165
Mapped reads (mapq ≥ 20)	897,213	959,151	959,663
Coverage with HQ bases (DP4 ≥ 5)	82%	82%	82%
Gene coverage (gene length ≥ 95%)	84%	81%	83%

Table S1. Whole genome sequencing (on the Roche-454 platform) and mapping statistics of parental, drug-sensitive *T. b. rhodesiense* STIB900 and its two drug-resistant derivatives STIB900-M and STIB900-P compared to the *T. b. brucei* TREU927 reference genome.
